# Test–Retest Reliability and Inter-Scanner Reproducibility of Improved Spinal Diffusion Tensor Imaging

**DOI:** 10.3390/diagnostics15162057

**Published:** 2025-08-16

**Authors:** Christer Ruff, Stephan König, Tim W. Rattay, Georg Gohla, Ulrike Ernemann, Benjamin Bender, Uwe Klose, Tobias Lindig

**Affiliations:** 1 Department of Diagnostic and Interventional Neuroradiology, Eberhard Karls-University Tuebingen, 72076 Tuebingen, Germany; 2 Center for Neurology, University Hospital Kiel, Arnold-Heller-Str 3, 24105 Kiel, Germany

**Keywords:** fractional anisotropy, mean diffusivity, radial diffusivity, spinal diffusion tensor imaging

## Abstract

**Background/Objectives**: Spinal diffusion tensor imaging (sDTI) remains a challenging method for the selective evaluation of key anatomical structures, like pyramidal tracts (PTs) and dorsal columns (DCs), and for reliably quantifying diffusion metrics such as fractional anisotropy (FA), radial diffusivity (RD), mean diffusivity (MD), and axial diffusivity (AD). This prospective, single-center study aimed to assess the reproducibility, robustness, and reliability of an optimized axial sDTI protocol, specifically intended for long fiber tracts. **Methods**: We developed an optimized Stejskal–Tanner sequence for high-resolution, axial sDTI of the cervical spinal cord at 3.0 T. Using advanced standardized evaluation and post-processing methods, we estimated DTI values for PTs, DCs, and AHs at the level of the second cervical vertebra. Reliability was evaluated through repeated measurements in 16 healthy volunteers and by comparing results from two 3.0 T scanners (Magnetom Skyra and Magnetom Prisma, Siemens Healthineers, Erlangen, Germany). Reproducibility was assessed using paired t-tests, intraclass correlation coefficients (ICCs), Bland–Altman analysis, and coefficients of variation (CVs). **Results**: The optimized sDTI protocol demonstrated high consistency for FA between test–retest sessions and across scanners. For the Skyra, the DC region showed the highest reliability (average ICC = 0.858) followed by the PT region (average ICC = 0.789). On the Prisma, the PT region reached an average ICC of 0.854, with the DC region at 0.758. Pooled inter-scanner data indicated good-to-excellent agreement, particularly in the PT region (average ICC = 0.860). FA CVs remained low (<10%) across all regions and scanners. RD showed good-to-excellent ICC values for PTs and DCs (average ICC for Skyra 0.642 and 0.769 and 0.926 and 0.830 for Prisma, respectively) but showed a higher CV between 14.6 and 19.4% for these two scanners. **Conclusions**: Improved sDTI offers highly reproducible FA measurements for all metrics with scanner independence, supporting its potential as a robust tool for detecting and monitoring spinal cord pathologies.

## 1. Introduction

Conventional MRI has long been utilized to visualize spinal cord disorders, offering excellent anatomic and morphologic information with high-resolution images. However, this technique is limited in detecting and quantifying microstructural changes in long spinal fiber tracts, which is crucial for understanding the etiology of conditions such as neurodegeneration, trauma, demyelinating, and inflammatory diseases, impacting clinical decision-making. Diffusion tensor imaging (DTI) is a promising technology for investigating the microstructure of spinal cord white matter, potentially providing quantitative measurements to detect abnormalities in otherwise normal-appearing structures [1,2]. Establishing reproducible and robust spinal DTI in clinical environments has the potential to further its use in disease states. DTI is, among other things, able to detect spinal cord abnormalities in multiple sclerosis [3], neuromyelitis optica [4], HIV myelopathy [5], and spondylotic myelopathy [6] when conventional MR imaging appears normal and, therefore, could potentially better guide treatment and predict outcomes, providing quantitative microstructural data that may show improved association with clinical presentation outcomes.

Despite its promise, spinal DTI (sDTI) is not yet commonly used in radiological practice due to several challenges [7,8,9,10,11,12]. Previous studies frequently use whole-cord ROIs derived from sagittal images or images with low axial in-plane resolution, which limits the granularity of the data, especially concerning the disease’s impact on white matter. This limitation is particularly significant given the inherent variations in DTI metrics between white and gray matter and the potential microstructural differences in how diseases affect these distinct tissues. Furthermore, these analyses frequently lack motion correction and involve the manual placement of ROIs, typically performed using a freehand technique. Such approaches introduce several technical challenges and limitations that can significantly impact the accuracy and reliability of the sDTI metrics obtained.

Furthermore, the small cross-sectional area of the spinal cord and magnetic field inhomogeneities from nearby vertebrae cause image distortions. Additionally, cerebrospinal fluid (CSF) pulsations, vascular pulsations, and respiratory motion generate significant artifacts in anterior–posterior and rostrocaudal directions. The limited signal-to-noise ratio (SNR) due to physiological artifacts and thermal noise is a significant concern in high in-plane resolution axial sDTI acquisition. Reliable tract-based evaluation methods of the white matter may suffer from reader-related variability and poor reproducibility, necessitating the development of automated or semiautomated spinal evaluation methods. A previous study by Lindig et al. successfully tested axially acquired sDTI on a patient population and revealed a pattern of robust changes in the pyramidal tracts and dorsal columns in hereditary spastic paraplegias (HSPs), along with unique features such as the ability to discriminate between normal aging and pathological conditions of the spinal cord when compared to a healthy control group [13]. What was lacking in this study, however, was an assessment of the reliability of measurement in repeated testing and the question of inter-scanner variability, both of which are of great relevance to clinical practice. The reproducibility of this method is important since it may be needed in follow-up studies, for example.

There are intrinsic limitations to the single-tensor DTI model when applied to regions with complex fiber architecture and high neuronal density, such as the anterior horns (AHs). Diffusion kurtosis imaging therefore might serve as a complementary microstructural probe for gray matter. This study, therefore, aimed to assess the reproducibility, robustness, and reliability of a potentially clinically viable, high-resolution sDTI method, especially for the selective evaluation of the pyramidal tracts (PTs) and dorsal columns (DCs) at the upper cervical level, where all long spinal tracts are present with the maximum number of fibers for all four extremities. The test–retest reliability, inter-scanner reproducibility, and robustness of the measurements were evaluated using a further adapted and more advanced semiautomated method for extracting tract-specific diffusion metrics. As demonstrated in the publication by Lindig et al., the estimation of the diffusion constant in the direction of the fiber pathways (axial diffusivity, AD) is challenging with this technique, due to the signal loss in the diffusion-weighted images with the b-values utilized in the aforementioned publication [13]. The primary objective of the applied technique is to ascertain the stability of the diffusion parameters in the transverse direction, especially in the long fiber tracts. 

## 2. Materials and Methods

### 2.1. Healthy Study Participants

This presented monocentric study was approved by the local institutional ethics committee (833/2016BO2, 115/2013BO2) and is conducted in accordance with the Declaration of Helsinki and its subsequent amendments. After obtaining informed consent, 16 consecutive healthy volunteers aged 21–27 were enrolled for evaluation. Exclusion criteria comprised any current or past neurological or psychiatric disorders, major medical comorbidities, and standard MRI contraindications. The final cohort included nine women (56%, median age 24 years) and seven men (44%, median age 23 years).

All images were obtained using 3.0 T MRI systems (MAGNETOM Skyra and MAGNETOM Prisma, Siemens Healthineers, Erlangen, Germany), each configured with a maximum gradient amplitude of 45 mT/m, a maximum slew rate of 200 mT/m/sec, and a 32-channel head coil. Test–retest scans and cross-scanner measurements were conducted sequentially on the same day.

### 2.2. Spinal DTI Protocol

Diffusion-weighted images were acquired using an optimized monopolar EPI sequence with double-spin-echo diffusion preparation at the level of the second cervical vertebra (C2) [14]. This location was selected because all long fiber tracts of the four extremities run through this height, and thoracic movement is less severe at the upper cervical cord. A single axial slice perpendicular to the cord’s long axis was acquired to distinguish between white and gray matter while minimizing partial volume effects. Cardiac gating compensated for cerebrospinal fluid (CSF) pulsation artifacts, with the optimal trigger delay visually determined on the corresponding b0 images. Acquisition parameters included a repetition time (TR) of 1500 ms, echo time (TE) of 82 ms, 128 × 128 matrix, 5 mm slice thickness, and a 100 × 100 mm^2^ field of view, resulting in an in-plane resolution of 0.78 × 0.78 mm^2^. Total scan time ranged from five to eight minutes, depending on the individually determined trigger setting. Diffusion-encoding gradients were applied along the directions e1 = (1,0,1), e2 = (−1,0,1), e3 = (0,1,1), e4 = (0,1,−1), e5 = (1,1,0), and e6 = (−1,1,0). These coordinates (x,y,z) are defined relative to the scanner frame of reference, with the spinal cord aligned parallel to the *z*-axis in the supine position. Additional technical details on the sDTI sequence are presented in Supplementary Table S1.

### 2.3. MRI Post-Processing and Data Analysis

**Figure 1:**
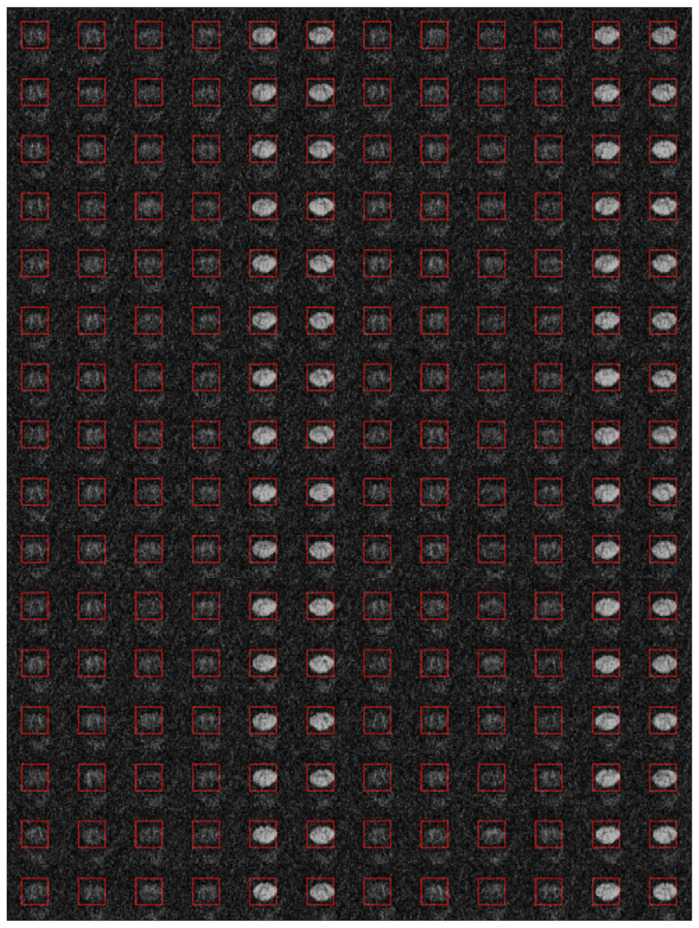
The axially acquired diffusion tensor imaging (DTI) slices of the spinal cord at the level of the second cervical vertebra (C2) were manually centered with the original field of view (FOV) and cropped to the intra-spinal space at the outer margins of the cerebrospinal fluid. The image shows all diffusion-weighted images of a healthy participant after the image section has been narrowed down and after an additional motion correction has been performed. During further post-processing of the DTI images, the image section was limited even further (indicated by the red image frame), which completely contained the spinal cord.

**Figure 2:**
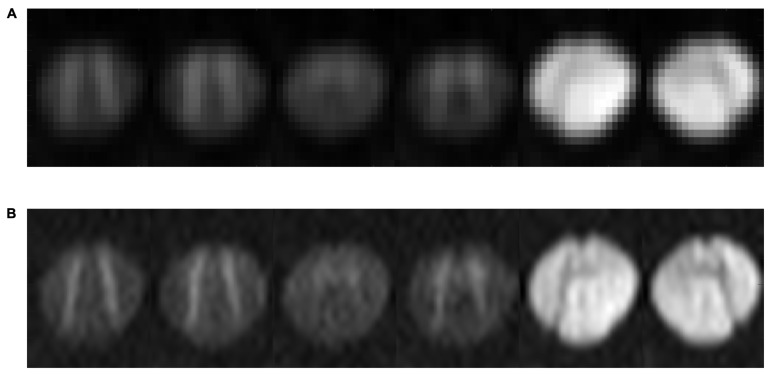
Images show the six diffusion directions of the axially acquired diffusion tensor imaging (DTI) slices of the spinal cord at the level of the second cervical vertebra (C2) after averaging over all 32 repetitions, once with the original resolution, with an in-plane resolution of 0.78 × 0.78 mm^2^ (**A**), and once after interpolation, with an in-plane resolution of 0.2 × 0.2 mm (**B**).

Diffusion-weighted images were analyzed offline using adapted routines written in Matlab R2015b (The MathWorks, Inc., Natick, MA, USA). The acquired slices with the original field of view (FOV) were manually centered and cropped to the intra-spinal space at the outer margins of the cerebrospinal fluid (Figure 1). After the exclusion of relevant eddy current-induced distortions by visual inspection, no eddy current correction was performed. Since scans of the upper cervical cord also suffer from breathing artifacts with slice displacement in the anterior–posterior direction, an automatic movement correction was added based on midsagittal intensity profiles aligned for each direction on the b0 image. DTI metric maps were interpolated to 0.2 × 0.2 mm^2^ before further post-processing to reduce partial volume effects (Figure 2). Figure 1 and Figure 2 show these post-processing steps with further explanations.

**Figure 3:**
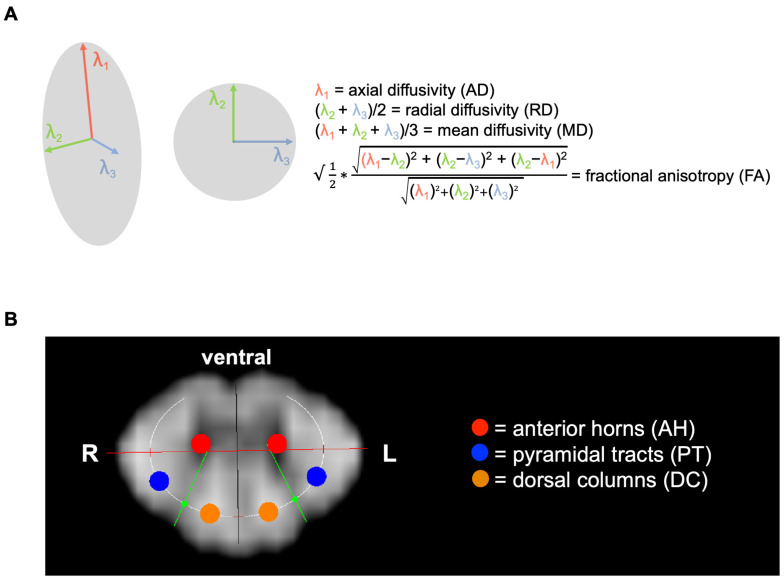
Schematic overview of diffusion tensor imaging (DTI) acquisition and resulting metrics. (**A**) Equations of DTI-derived parameters, including fractional anisotropy (FA), axial diffusivity (AD; diffusion along the axon, λ1), radial diffusivity (RD; diffusion perpendicular to the axon, averaging λ2 and λ3), and mean diffusivity (MD; mean diffusion over λ1, λ2, and λ3). (**B**) A single 5 mm axial slice (in-plane resolution 0.78 × 0.78 mm^2^) was acquired at the level of the second cervical vertebra (C2) from a healthy participant. In-plane spectral fat suppression and two saturation bands along the phase-encoding direction were used to prevent folding artifacts, with phase encoding aligned anterior-to-posterior in the body. Image shows representative fractional anisotropy (FA) maps, illustrating the semiautomated, bilateral region-of-interest (ROI) evaluation for the pyramidal tracts (PTs, blue), dorsal columns (DCs, orange), and anterior horns (AHs, red). An ellipsoid was fitted to the individual FA map through iterative adjustment of its major and minor axes and the posterior horns’ exit angles (green).

The basic diffusion parameters, axial diffusivity (AD) and radial diffusivity (RD), were created by fitting a tensor model to the raw diffusion data. The thereof-derived diffusion measures, fractional anisotropy (FA) and mean diffusivity (MD), were calculated. MD represents diffusion independent of the orientation of tissue (MD = (λ1 + λ2 + λ3)/3), AD reveals diffusion in the longitudinal direction of the WM fibers (AD = λ1), RD represents diffusion in the perpendicular directions of the WM fibers (RD = (λ1 + λ2)/2), and FA measures the degree of the orientation of water proton diffusivity in tissues. The calculation of FA follows a more complex formula in which the individual diffusion directions do not have such a high individual influence (Figure 3A). In a previous study by Lindig et al., RD and AD values were normalized to the maximum values within the cropped region of the intra-spinal space, resulting in relative values [13]. In contrast, this study uses the actual calculated values without normalization, which results in a different level on the *y*-axis and is therefore more comparable to other DTI studies. An adjusted ellipsoid was placed onto each healthy participant’s individual FA image of the spinal cord during a consensus reading (Figure 3B). For an evaluation of the profile along the drawn elliptical circle, the angular sampling was 600 points for the whole circle, i.e., 0.6°. The long and short axis of the ellipsoid, together with the exit angles of the posterior horns, were adjusted to the plotted profile of the ellipsoid. In the analysis, the mean DTI values of the left- and right-running fiber tracts were calculated for a total of six ROIs (1.05 mm in diameter). In the publication, the mean values of two matching ROIs situated in PTs, DCs, and AHs are referred to as FA, RD, MD, and AD. Individual mean DTI values of healthy study participants’ PTs, DCs, and AHs were compared between tests and scanners.

### 2.4. Statistical Analysis

Statistical analyses were performed using SPSS Statistics Version 30 (IBM, Armonk, NY, USA). A paired-samples t-test evaluated potential differences in participant parameter values between tests and between scanners, with a corrected *p*-value < 0.05 considered statistically significant. Cohen’s d was calculated for each right- and left-sided fiber tract across scanners, interpreted as follows: small |d| = 0.20–0.49; medium |d| = 0.50–0.79; and large |d| ≥ 0.80.

The single and average within-participant intra-class correlation coefficients (ICCs) of DTI metrics were used to assess the reliability of repeated scans. The single ICC reflects scenarios in which each measurement is acquired only once (common in clinical practice), while the average ICC pertains to situations where the same region is measured twice and the mean of the two measurements is taken. The ICC is calculated as [true variance/(true variance + error variance)] representing the proportion of variation due to the participant’s “true” error-free values [15].

The reliability of test–retest DTI metrics was evaluated using a single-rater, consistent-agreement, two-way, mixed-effects model of intra-class correlation (ICC) for absolute agreement [16]. Higher ICC values indicate stronger reliability, as described by Shrout and Fleiss, Cicchetti, and Koo et al. Table 1 presents both single and average ICC estimates [15,16,17]. According to Koo et al., emphasis should be placed on the 95% confidence interval of the ICC estimate rather than the ICC value alone [16].

For measurement errors, the coefficient of variation (CV) was measured for each DTI variable to clarify the relative variability of measurement: CV = [the within-participant standard deviation]/within-participant mean × 100%. A CV of <10% is considered acceptable, indicating that the dependent variable has a relatively small variation [18]. A CV between 11% and 20% is considered adequate and indicates a moderate variation. A CV > 20% represents high variability [19]. To further assess agreement and variability between measurements, Bland–Altman (BA) plots with 95% confidence intervals (CIs) were generated by plotting the average measurement ([visit1 + visit2]/2) against the measurement difference (visit1–visit2). Smaller differences indicate better agreement, with a perfect Bland–Altman plot represented by a horizontal line at y = 0, suggesting no difference between test and retest measurements. GraphPad Prism version 10.5.0 was used to create the plots (GraphPad Software Inc., Dotmatics, Boston, MA, USA).

## 3. Results

**Figure 4:**
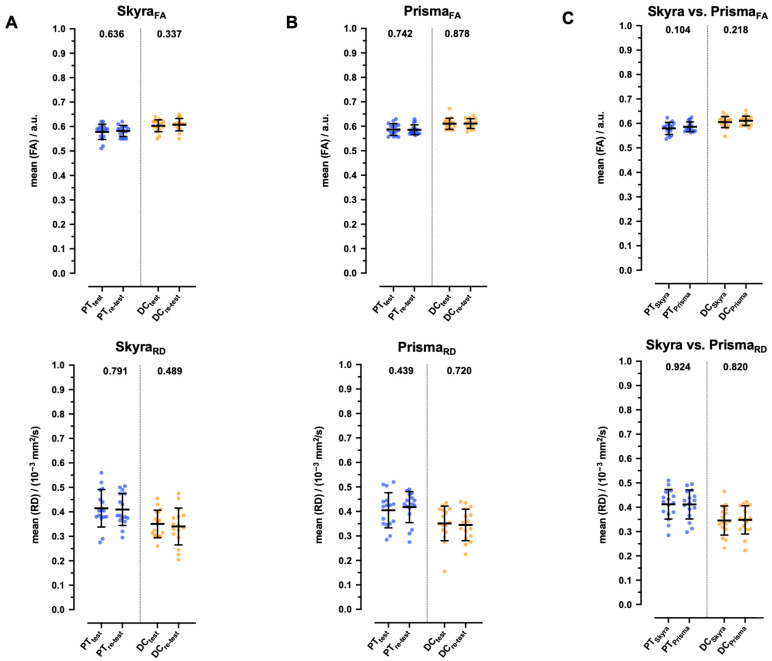
Differences in fractional anisotropy (FA) and radial diffusivity (RD) between test and retest in healthy study participants for Magnetom Skyra and Magnetom Prisma. Boxplots showing group differences in FA and RD among healthy study participants (*n* = 16) in pyramidal tracts (PTs) and dorsal columns (DCs) for Magnetom Skyra (**A**) and Magnetom Prisma (**B**) and between pooled data of both scanners (**C**). Data is shown as mean and standard deviation (SD). *p*-values are indicated.

**Figure 5:**
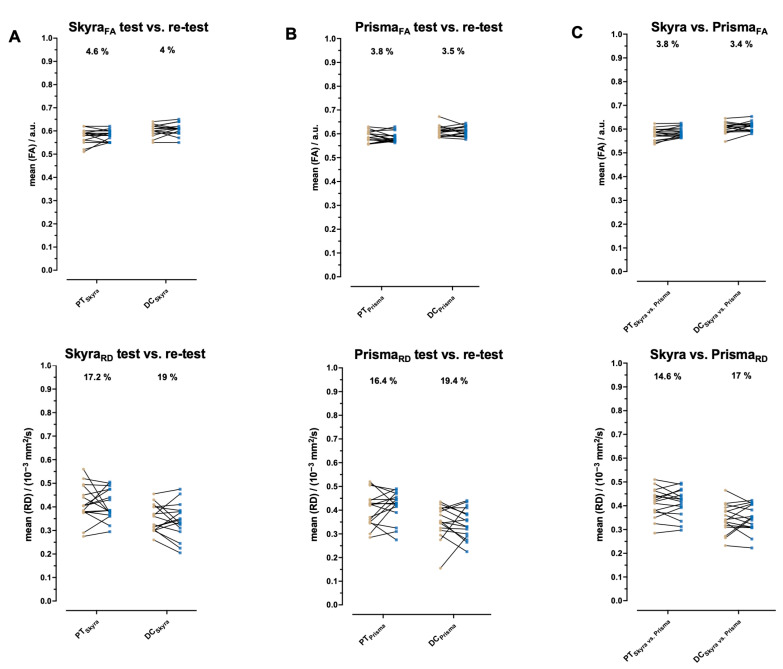
Differences in fractional anisotropy (FA) and radial diffusivity (RD) between test and retest in healthy study participants for Magnetom Skyra and Magnetom Prisma. Plots showing between-group differences in FA and RD among healthy study participants (*n* = 16) in pyramidal tracts (PTs) and dorsal columns (DCs) for Magnetom Skyra (**A**) and Magnetom Prisma (**B**) and between pooled data of both scanners (**C**), with connecting lines between test and retest. Within participants, the coefficient of variation (CV %) is indicated.

**Figure 6:**
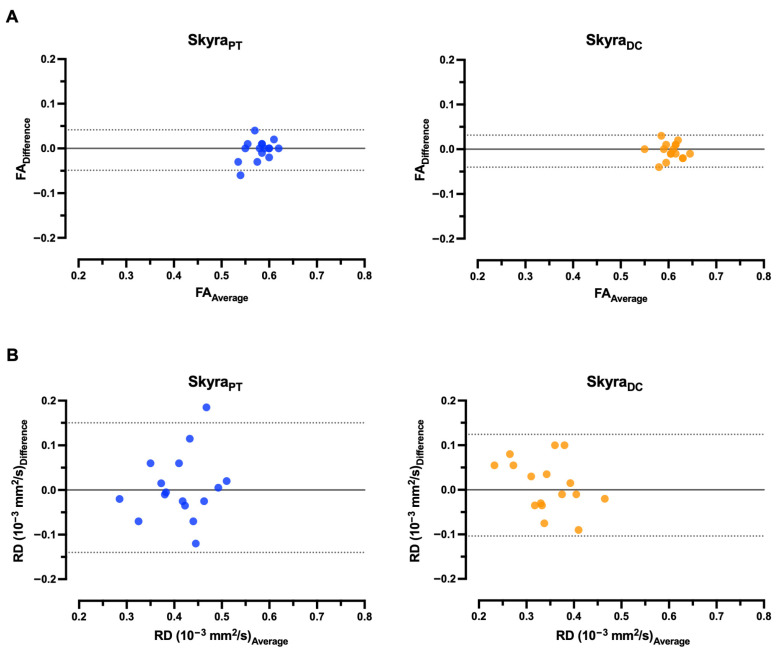
Bland–Altman plots comparing within-participant test–retest values for Magnetom Skyra, exhibiting the difference (solid black lines) and Limit-of-Agreement 95% confidence interval (dotted black lines) in pyramidal tracts (PTs) and dorsal columns (DCs) in each healthy study participant (*n* = 16). The differences in diffusivity metrics, fractional anisotropy (FA, (**A**)) and radial diffusivity (RD, (**B**)), for pairs of scans are plotted against the average of the diffusivity metrics.

**Figure 7:**
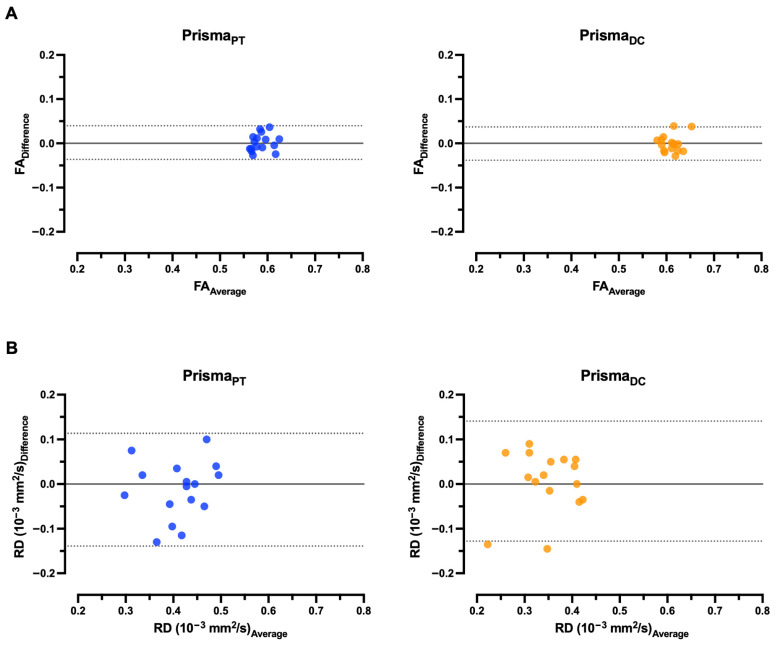
Bland–Altman plots comparing within-participant test–retest values for Magnetom Prisma, exhibiting the difference (solid black lines) and Limit-of-Agreement 95% confidence interval (dotted black lines) in pyramidal tracts (PTs) and dorsal columns (DCs) in each healthy study participant (*n* = 16). The differences in diffusivity metrics, fractional anisotropy (FA, (**A**)) and radial diffusivity (RD, (**B**)), for pairs of scans are plotted against the average of diffusivity metrics.

**Figure 8:**
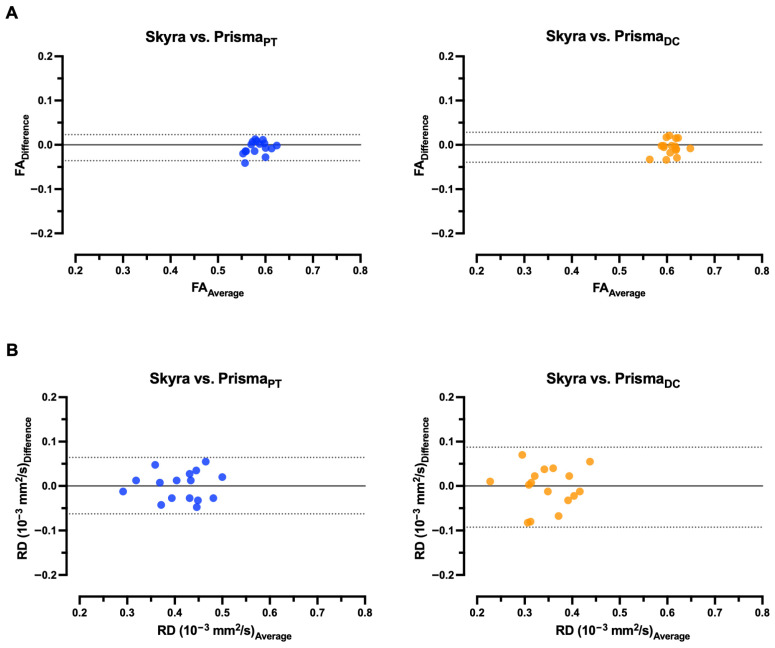
Bland–Altman plots comparing within-participant pooled test–retest values for Magnetom Skyra and Magnetom Prisma, exhibiting the difference (solid black lines) and Limit-of-Agreement 95% confidence interval (dotted black lines) in pyramidal tracts (PTs) and dorsal columns (DCs) in each healthy study participant (*n* = 16). The differences in diffusivity metrics, fractional anisotropy (FA, (**A**)) and radial diffusivity (RD, (**B**)), for pairs of scans are plotted against the average of diffusivity metrics.

None of the 16 healthy study participants were excluded. Detailed results of the diffusivity parameters FA, RD, MD, and AD for both the individual measurements and the averaged (pooled) values of the repeated measurements for Magnetom Skyra and Magnetom Prisma are shown in Table 2, Table 3 and Table 4. Since FA and RD showed the best matches, they are the focus of the following analysis, and their graphical representations are presented in Figure 4, Figure 5, Figure 6, Figure 7 and Figure 8. The parameters MD and AD are also illustrated graphically, but in Supplementary Figure S1.

### 3.1. Evaluation of sDTI Parameters in Healthy Study Participants

For both the Magnetom Skyra and Magnetom Prisma scanners, there were no significant changes between test and retest measurements in the mean effect sizes for FA and RD (values are given below in units of 10^−3^ mm^2^/s). Additionally, no significant differences were observed between these pooled values from both devices across all measured regions (Table 2, Table 3 and Table 4, Figure 4).

In PTs, the mean FA values for Skyra_FA_ between the test and retest were 0.578 ± 0.031 vs. 0.581 ± 0.023 (*p* = 0.636), and for Prisma_FA_, they were 0.587 ± 0.024 vs. 0.585 ± 0.020 (*p* = 0.742). In DCs, Skyra_FA_ values were 0.603 ± 0.023 vs. 0.607 ± 0.026 (*p* = 0.337), and Prisma_FA_ values were 0.610 ± 0.023 vs. 0.611 ± 0.020 (*p* = 0.878).

Regarding RD, in the PT region, Skyra_RD_ values between the test and retest were 0.415 ± 0.076 vs. 0.410 ± 0.065 (*p* = 0.791), and Prisma_RD_ values were 0.405 ± 0.072 vs. 0.418 ± 0.063 (*p* = 0.439). In the DC region, Skyra_RD_ values were 0.351 ± 0.056 vs. 0.340 ± 0.075 (*p* = 0.489), and Prisma_RD_ values were 0.351 ± 0.071 vs. 0.345 ± 0.064 (*p* = 0.720).

The medium effect size of FA observed in PTs of pooled data between Skyra_FA_ and Prisma_FA_ is 0.580 ± 0.025 vs. 0.586 ± 0.020 (*p* = 0.104), in DCs, 0.605 ± 0.023 vs. 0.611 ± 0.019 (*p* = 0.219), and in AHs, 0.414 ± 0.033 vs. 0.404 ± 0.033 (*p* = 0.239). The medium effect size of RD (10^−3^ mm^2^/s) observed in PTs of pooled data between Skyra_RD_ and Prisma_RD_ is 0.412 ± 0.061 vs. 0.411 ± 0.060 (*p* = 0.924), and in DCs, 0.345 ± 0.060 vs. 0.348 ± 0.058 (*p* = 0.82).

### 3.2. Within-Participant Intra-Class Correlation

Single and average measured ICCs of DTI metrics for Magnetom Skyra (Table 2), Magnetom Prisma (Table 3), and their pooled data comparison (Table 4), respectively, showed variable reliability, depending on the DTI value and localization.

Skyra_FA_ demonstrated moderate-to-good agreement for single ICC values according to Koo et al., and good-to-excellent agreement according to Cicchetti. Meanwhile, the average ICC showed good-to-excellent consistency per Koo et al. and was rated as excellent by Cicchetti, with the DC region yielding the highest consistency (single/average ICC = 0.751/0.858), followed by the PT region (single/average ICC = 0.652/0.789). Prisma_FA_ exhibited moderate agreement for single ICCs based on Koo et al.’s criteria and moderate-to-good agreement according to Cicchetti. In contrast, the average ICC was rated as moderate-to-good by Koo et al. and good-to-excellent by Cicchetti, with the PT region achieving the highest consistency (single/average ICC = 0.630/0.854), followed by the DC region (single/average ICC = 0.610/0.758).

Skyra_RD_ showed moderate agreement for single ICCs according to Koo et al. and moderate-to-good agreement according to Cicchetti. The average ICC was rated as moderate-to-good by Koo et al. and good-to-excellent by Cicchetti, with the DC region exhibiting the highest consistency (single/average ICC = 0.624/0.769), followed by the AH region (single/average ICC = 0.550/0.709). Prisma_RD_ displayed poor-to-moderate agreement for single ICCs based on Koo et al.’s criteria and moderate agreement according to Cicchetti. The average ICC was rated as moderate-to-good by Koo et al. and good by Cicchetti, with the PT region achieving the highest consistency (single/average ICC = 0.553/0.712), followed by the DC region (single/average ICC = 0.498/0.665).

The single and average ICCs of DTI metrics for the pooled data between Skyra_FA_ and Prisma_FA_ indicated good agreement according to Koo et al., and excellent agreement according to Cicchetti. The PT region showed the highest consistency (single/average ICC = 0.755/0.860), followed by the DC region (single/average ICC = 0.653/0.790). For the pooled data between Skyra_RD_ and Prisma_RD_, the average ICCs demonstrated good-to-excellent agreement, with the PT region exhibiting the highest consistency (single/average ICC = 0.863/0.926), followed by the DC region (single/average ICC = 0.709/0.830).

### 3.3. Within-Participant Variation

The within-participant variations (CV %) are shown in the corresponding Table for Magnetom Skyra (Table 2), Magnetom Prisma (Table 3), and the pooled data between both scanners (Table 4), as well as in Figure 5 for FA and RD. The FA, MD, and AD CVs were low and acceptable (<10%) for all examined localizations and scanners, indicating that the dependent variable has a relatively small variation. For Skyra_FA_, the CV was 4.6% in PTs, 4.0% in DCs, and 8.6% in AHs. For Prisma_FA_, the CV was 3.8% in PTs, 3.5% in DCs, and 9.3 % in AHs. For the pooled data analysis between both scanners, the CV for FA was 3.8% in PTs, 3.4% in DCs, and 8.1% in AHs, showing high reproducibility. The CV of RD, however, was only adequate, ranging from 11.2% to 19.4% for both scanners, indicating a moderate variation except for the pooled data analyses for AH (9.6%).

### 3.4. Bland–Altman (BA) Analysis Between Test and Retest

Bland–Altman (BA) plots for within-participant values of FA and RD in PTs and DCs are shown in Figure 6 for Magnetom Skyra, Figure 7 for Magnetom Prisma, and Figure 8 for the inter-scanner evaluation. The mean differences (D) of FA and RD for the two scans were minor between the scans, indicating good agreement between visits for each DTI parameter. For example, the 95% CI for mean differences in these metrics overlaps zero in contrast to MD and AD, where this does not apply to individual values in Magnetom Prisma and the pooled data comparison between the two scanners for all regions examined (Table 3 and Table 4). The 95% CI for FA is narrower than for RD across both scanners and all examined localizations. Data points outside the 95% CI correspond to a significant deviation between the test and retest. For Skyra_FA_ and Skyra_RD_, this is the case for one data point in PTs. For Prisma_FA_, this is the case for one data point in DCs, and for Prisma_RD_, for two data points in DCs. Regarding the pooled data analysis between both scanners, this is the case for one participant in PTs.

## 4. Discussion

This study presents the first comprehensive evaluation of test–retest and inter-scanner reproducibility for semiautomated high-resolution axial DTI of the cervical spinal cord. Reproducible DTI measurements are essential for effectively evaluating progressive cervical spinal cord diseases, guiding treatment strategies, and monitoring disease stability over time. Our findings indicate that FA demonstrates a higher level of agreement between repeated measurements compared to other DTI parameters, underscoring the reliability of this imaging modality and parameter also in an inter-scanner setting.

Segmentation methods applied to the cervical spinal cord significantly impact the reproducibility of DTI metrics. Many studies have utilized manual ROIs, which are prone to bias, labor-intensive, and do not offer atlas-based segmentation assessments or tract-specific information [20,21,22,23,24,25,26]. As the cord volume decreases between C1 and T1, manual ROIs at each disc and mid-level of a cervical vertebral body are susceptible to contamination from CSF or adjacent tissue structures [24]. ROIs encompassing the entire cord, including both WM and GM, often provide limited detail, especially concerning disease effects on WM regions, given the differences in DTI indices between WM and GM and potential microstructural changes [27]. In spinal cord diseases like cervical spondylotic myelopathy, low-spatial-resolution DTI images and atrophied spinal cords make accurate manual ROI delineation challenging. In contrast, a few studies have applied automatic segmentation methods [26,27,28,29,30]. This approach offers tract-based indices and robust readouts from different ROIs, validated by reproducibility analyses that can provide increased specificity regarding clinical damage compared to analyses of the entire cord [27].

The number of diffusion gradient directions selected may influence the reproducibility of DTI metrics. By et al. evaluated this effect by comparing quantitative DTI results obtained using 6, 15, and 32 directions with automatically segmented WM and GM ROIs between C2 and C5 [28]. They found that normalized Bland–Altman differences between repeated scans were below 14% for all metrics, with the largest discrepancies observed in the 32-direction data. With regard to the number of directions, it should be noted that the good reproducibility observed in our study is already evident with the six directions used. Therefore, it is not absolutely necessary to add further measuring directions and therefore a longer measuring time. This is an important point for potential clinical follow-up studies with patients. Various studies have reported normalized Bland–Altman differences in DTI metrics using multiple ROIs [20,25,28]. In these studies, the inter-reader normalized differences ranged from 1.89% to 2.06%, while intra-reader assessments ranged from 2.38% to 4.54% for all DTI metrics except for RD in Smith et al., which showed a higher percentage (difference  =  8.44%), attributed to a strong dependency on image resolution and an enhanced signal-to-noise ratio due to sequence parameters [20]. In our study, RD exhibited worse reproducibility than FA regarding the Limits of Agreement in Bland–Altman analysis and the coefficient of variation.

Previous studies have examined the reproducibility of spinal cord DTI, primarily using manual ROI assessments. Brander et al. analyzed 40 healthy controls and found excellent intrareader and good interreader agreement for whole-cord FA and apparent diffusion coefficient (ADC) values using manual ROIs and tractography-based analysis [31]. Mulcahey et al. evaluated the scan–rescan reproducibility of DTI metrics in 10 pediatric patients with chronic spinal cord injury, reporting ICCs ranging from 0.50 to 0.95 across various DTI metrics, depending on cervical spinal cord level (0.50–0.89 for FA, 0.80–0.95 for MD, 0.82–0.94 for AD, and 0.82–0.94 for RD) [23]. Smith et al. assessed scan–rescan and interreader reproducibility in nine healthy volunteers, finding no significant differences between readers or scans, with normalized Bland–Altman differences ranging from 1.89% to 4.54% [20]. These studies predominantly utilized manual ROI techniques, which are labor-intensive, cumbersome, and cannot provide tract-specific information.

Technical factors such as FOV, resolution, and motion correction techniques significantly influence the quality of spinal DTI data. The sDTI sequence used in this study represents a substantial advancement over prior methodologies by employing a low FOV, high axial in-plane resolution, and motion correction. This approach contrasts previous studies that often relied on sagittal acquisitions with low axial resolution and manual ROI placement on axial FA maps, practices that increase the risk of inaccurate results due to the imprecision of freehand ROI placement [31,32,33]. Data processing steps, including motion correction and spinal cord segmentation, are essential for achieving robust and automated interpretation of morphometric and multiparametric MR images, minimizing artifacts, ensuring data accuracy, and facilitating the extraction of meaningful and reliable metrics.

Motion correction and spinal cord segmentation are crucial for robust and automated interpretation of MR images. Motion correction compensates for patient movement during scanning, a significant challenge in spinal cord imaging due to the cord’s small size and sensitivity to minor movements, ensuring images accurately reflect underlying anatomy and pathology. Spinal cord segmentation isolates the cord from surrounding tissues, allowing precise measurement and analysis of morphology and imaging parameters, essential for detailed assessment of structural and functional changes necessary for clinical diagnosis and research [1]. This study employed a semi-automatic methodology, utilizing a bilateral, geometry-based ROI approach to objectively segment WM and GM structures, including the pyramidal tracts, dorsal columns, and anterior horns. Although full automation has not yet been achieved, we have already adopted several measures to limit subjective influence. In particular, we applied an elliptical mask that conforms to the spinal cord contour and defined regions of interest based on the voxel-wise intensity profile. This effort can, therefore, be regarded as an advanced, semi-automated interim solution. The findings demonstrate that this evaluation technique for sDTI is both technically feasible and reliable for obtaining regionally selective measurements of FA and RD. This approach aligns with similar semi-automatic methods previously assessed and validated by Xu et al. and Klawiter et al., supporting its applicability and robustness in sDTI analysis [1,34]. Geometry-based ROI segmentation techniques have advantages over tractography or fuzzy-logic approaches [35,36].

A reliable imaging method capable of objectively quantifying pathological changes in the long fiber tracts of the central nervous system is urgently needed. sDTI has the potential to correlate imaging findings with the duration and severity of clinical disability. Alterations in sDTI parameters may precede morphological changes like volume reduction, indicating microstructural alterations before they become visible through conventional imaging techniques. Thus, sDTI could fill a critical gap in clinical and research applications by enabling monitoring of disease progression, assessing treatment responses, and guiding therapeutic decisions. Our findings emphasize the feasibility and reliability of column-specific analysis in evaluating spinal cord disorders using DTI. Traditional whole-cord DTI analysis often fails to capture subtle abnormalities within individual WM columns, potentially overlooking minor microstructural impairments. With its higher in-plane resolution, our method addresses this limitation by reducing partial volume effects, providing more detailed and accurate assessments.

Further enhancements in sDTI could be achieved by implementing advanced acquisition techniques, such as selective volume imaging (e.g., ZOOMit), which offers faster acquisition, higher resolution, and fewer susceptibility artifacts and distortions [37]. Transitioning from the semi-automatic motion correction and spinal cord segmentation used in this study to fully automated processes could further improve clinical usability by reducing potential rater bias and streamlining the workflow. Despite these areas for improvement, our results indicate a promising path toward broader clinical adoption of sDTI. We demonstrate that axial sDTI, when combined with motion correction and advanced post-processing strategies, enables robust and reliable FA measurements, making it ready for clinical application. These results may encourage the exploration of sDTI as a selective biomarker in diseases involving spinal cord pathology, potentially expanding its role in diagnostic and therapeutic contexts.

This study has several limitations that should be considered when interpreting the findings. First, the study involved a relatively small cohort of 16 healthy participants. While this provides initial insights and is in line with other sDTI studies, it underscores the need for future research with larger, more diverse cohorts to validate and generalize these results more broadly. Second, the study’s inclusion of a homogeneous age group, consisting exclusively of young, healthy individuals, introduces potential confounding factors. These factors may influence the outcomes and limit the applicability of the results to older or, in general, broader, more varied populations. Repeatability might deteriorate in older participants, principally because of motion and respiratory artifacts rather than intrinsic shortcomings of the DTI sequence itself. However, this present work is a technical reproducibility study, not a feasibility trial. It expands upon our earlier investigation, which enrolled > 100 healthy adults aged 18–65 years and individuals with HSP. That study showed that spinal DTI metrics remain stable across the age span between 18 and 65 years and that long-tract pathology can be robustly detected in diseased patients, thereby demonstrating the method’s applicability to older cohorts [13]. Third, the study utilized only six diffusion directions for fiber tracking and advanced diffusion parameters. Using a higher number of directions could influence the variability and reliability of the results. Therefore, future studies should consider incorporating more diffusion directions to improve the robustness of the findings [38]. Our previous work in HSP demonstrated that a six-direction scheme combined with multiple signal averages was sufficient to detect pathological change and, importantly, kept acquisition times within a clinically practical window [13]. Fourth, the study did not explore the potential benefits of using more advanced acquisition techniques, such as compressed sensing. These techniques have been shown to accelerate DTI and may improve clinical applicability. Incorporating such techniques in future research could potentially enhance the speed and improve the clinical use and accuracy of DTI scans [39,40]. A comprehensive, head-to-head comparison between different DTI techniques would provide additional insights. However, the breadth of such an endeavor—encompassing multiple protocols, optimization parameters, and an adequately powered sample—would extend well beyond the scope of the present manuscript, whose primary aim is to establish the reproducibility of our current technique. Fifth, the focus of this study was limited to the cervical spine, partly due to the relatively wide transverse diameter of the cervical myelon providing access to the long fiber tracts of all four extremities. However, this restriction means the findings may not apply to other spinal cord regions, such as the lower thoracic spinal cord. A factor that might impact the reproducibility of DTI metrics is the cervical spinal cord level examined, as observed in other studies where DTI characteristics were acquired at different spinal cord levels and reported different ICC values [24,26,41]. Future studies should expand the scope to include these areas to provide a more comprehensive understanding of spinal DTI and its applicability. Lastly, the study used MRI scans from only two scanners produced by the same manufacturer and located within a single university hospital. This limitation reduces the generalizability of the results. “True” inter-scanner reproducibility—i.e., across platforms from different manufacturers—cannot be realized within a single-centered setting. Nevertheless, the present single-center, single-vendor design fulfills our primary aim to establish a rigorous reproducibility benchmark that will underpin forthcoming longitudinal investigations. Future research should involve more extensive studies using a multi-center approach to address this. This would help validate the findings and establish the broader reproducibility of the sDTI technique used in this study.

## 5. Conclusions

This study represents further advancement in the ongoing effort to integrate spinal cord diffusion tensor imaging into clinical practice by establishing FA as the most robust parameter for spinal cord assessment. FA exhibits high column-specific test–retest reliability and inter-scanner reproducibility. The technique’s robustness across different scanning environments and its ability to yield consistent results are crucial for its adoption in clinical settings, where it could impact patient care by providing clinicians with a reliable tool for decision-making. Its application could be used in the assessment and management of conditions that affect long fiber tracts, such as HSP, amyotrophic lateral sclerosis (ALS), Friedreich’s ataxia, spinal cord ischemia, spinal tumors, and multiple sclerosis by providing objective and quantifiable metrics.

## Figures and Tables

**Table 1:** Interpretation of the classification of single and average intraclass correlation coefficient (ICC) values indicative for reliability as suggested by Cicchetti and Koo et al. [15,16].

ICC Estimate Based on the 95% CI According to		ICC Classification
Cicchetti	Koo et al.	
<0.4	<0.5	Poor
0.4 < ICC < 0.59	0.5 < ICC < 0.75	Moderate
0.60 < ICC < 0.74	0.75 < ICC < 0.9	Good
0.75 < ICC < 1	>0.9	Excellent

CI = confidence interval; ICC = intraclass correlation coefficient.

**Table 2:** Results of quantitative measurements of anisotropy and diffusivity in healthy participants (*n* = 16) for Magnetom Skyra.

Parameter	Magnetom Skyra				Bland–Altman Analysis		Within-Participant Coefficient of Variation CV %	Intraclass Correlation Coefficient (ICC)	
	Test ± SD (CI 95%)	Retest ± SD (CI 95%)	Pooled ± SD (CI 95%)	*p* Value Test vs. Retest	Difference (D)	LOA		Single (CI 95%)	Average (CI 95%)
FA									
PT	0.578 ± 0.031 (0.562–0.595)	0.581 ± 0.023 (0.569–0.593)	0.580 ± 0.025 (0.567–0.593)	0.636	−0.003 ± 0.023 (−0.015 to 0.010)	−0.048 to 0.042	4.6	0.652 (0.243–0.863)	0.789 (0.391–0.927)
DC	0.603 ± 0.023 (0.591–0.615)	0.607 ± 0.026 (0.594–0.621)	0.605 ± 0.023 (0.593–0.617)	0.337	−0.004 ± 0.017 (−0.013 to 0.005)	−0.038 to 0.029	4.0	0.751 (0.433–0.905)	0.858 (0.604–0.95)
AH	0.417 ± 0.037 (0.397–0.437)	0.411 ± 0.034 (0.393–0.429)	0.414 ± 0.033 (0.396–0.432)	0.372	0.006 ± 0.027 (−0.008 to 0.020)	−0.046 to 0.058	8.6	0.726 (0.384–0.894)	0.841 (0.555–0.944)
RD (10^−3^ mm^2^/s)									
PT	0.415 ± 0.076 (0.374–0.455)	0.410 ± 0.065 (0.375–0.444)	0.412 ± 0.061 (0.380–0.444)	0.791	0.005 ± 0.074 (−0.034 to 0.044)	−0.140 to 0.150	17.2	0.472 (−0.032–0.78)	0.642 (−0.067–0.876)
DC	0.351 ± 0.056 (0.321–0.381)	0.340 ± 0.075 (0.300–0.380)	0.345 ± 0.060 (0.314–0.377)	0.489	0.010 ± 0.058 (−0.021 to 0.041)	−0.104 to 0.124	19.0	0.624 (0.205–0.85)	0.769 (0.34–0.919)
AH	0.649 ± 0.067 (0.613–0.684)	0.643 ± 0.078 (0.601–0.684)	0.646 ± 0.064 (0.612–0.680)	0.726	0.006 ± 0.070 (−0.031 to 0.044)	−0.131 to 0.144	11.2	0.550 (0.079–0.817)	0.709 (0.146–0.899)
MD (10^−3^ mm^2^/s)									
PT	1.131 ± 0.064 (1.097–1.165)	1.130 ± 0.072 (1.091–1.169)	1.130 ± 0.052 (1.102–1.158)	0.977	0.001 ± 0.087 (−0.046 to 0.047)	−0.170 to 0.171	6.0	0.194 (−0.357–0.627)	0.324 (−1.111–0.771)
DC	1.119 ± 0.065 (1.084–1.154)	1.117 ± 0.083 (1.072–1.161)	1.118 ± 0.066 (1.083–1.153)	0.9	0.002 ± 0.069 (−0.035 to 0.039)	−0.133 to 0.137	6.6	0.592 (0.138–0.837)	0.744 (0.243–0.911)
AH	1.064 ± 0.065 (1.030–1.099)	1.038 ± 0.072 (0.999–1.076)	1.051 ± 0.054 (1.022–1.080)	0.222	0.027 ± 0.083 (−0.018 to 0.071)	−0.137 to 0.190	6.5	0.253 (−0.232–0.649)	0.403 (−0.604–0.787)
AD (10^−3^ mm^2^/s)									
PT	2.561 ± 0.165 (2.473–2.649)	2.571 ± 0.167 (2.482–2.660)	2.566 ± 0.147 (2.488–2.645)	0.792	−0.010 ± 0.154 (−0.092 to 0.072)	−0.312 to 0.291	6.5	0.586 (0.132–0.834)	0.739 (0.233–0.91)
DC	2.655 ± 0.206 (2.545–2.765)	2.670 ± 0.195 (2.566–2.773)	2.662 ± 0.191 (2.561–2.764)	0.64	−0.015 ± 0.126 (−0.082 to 0.052)	−0.261 to 0.231	7.5	0.812 (0.541–0.93)	0.896 (0.702–0.964)
AH	1.893 ± 0.148 (1.814–1.972)	1.831 ± 0.128 (1.763–1.899)	1.862 ± 0.118 (1.799–1.925)	0.104	0.063 ± 0.145 (−0.015 to 0.140)	−0.221 to 0.346	7.4	0.425 (−0.026–0.745)	0.596 (−0.053–0.854)

FA = fractional anisotropy; RD = radial or perpendicular diffusivity; MD = mean diffusivity; AD = axial diffusivity; PTs = pyramidal tracts; DCs = dorsal columns; AHs = anterior horns; CI = confidence interval; CV = coefficient of variation; ICC = intraclass correlation coefficient; LOAs = Limits of Agreement; SD = standard deviation.

**Table 3:** Results of quantitative measurements of anisotropy and diffusivity in healthy participants (*n* = 16) for Magnetom Prisma.

Parameter	Magnetom Prisma				Bland–Altman Analysis		Within-Participant Coefficient of Variation CV%	Intraclass Correlation Coefficient (ICC)	
	Test ± SD (CI 95%)	Retest ± SD (CI 95%)	Pooled ± SD (CI 95%)	*p* Value Test vs. Retest	Difference (D)	LOA		Single (CI 95%)	Average (CI 95%)
FA									
PT	0.587 ± 0.024 (0.574–0.600)	0.585 ± 0.020 (0.575–0.596)	0.586 ± 0.020 (0.576–0.597)	0.742	0.002 ± 0.019 (−0.009 to 0.012)	−0.036 to 0.040	3.8	0.630 (0.203–0.854)	0.773 (0.337–0.921)
DC	0.610 ± 0.023 (0.598–0.622)	0.611 ± 0.020 (0.600–0.622)	0.611 ± 0.019 (0.600–0.621)	0.878	−0.001 ± 0.019 (−0.011 to 0.010)	−0.038 to 0.037	3.5	0.610 (0.167–0.845)	0.758 (0.287–0.916)
AH	0.407 ± 0.039 (0.386–0.428)	0.400 ± 0.036 (0.381–0.420)	0.404 ± 0.033 (0.386–0.421)	0.459	0.007 ± 0.036 (−0.012 to 0.026)	−0.064 to 0.077	9.3	0.549 (0.093–0.815)	0.709 (0.17–0.898)
RD (10^−3^ mm^2^/s)									
PT	0.405 ± 0.072 (0.367–0.443)	0.418 ± 0.063 (0.384–0.452)	0.411 ± 0.060 (0.380–0.443)	0.439	−0.013 ± 0.064 (−0.047 to 0.022)	−0.139 to 0.113	16.4	0.553 (0.1–0.817)	0.712 (0.182–0.899)
DC	0.351 ± 0.071 (0.314–0.389)	0.345 ± 0.064 (0.311–0.379)	0.348 ± 0.058 (0.317–0.379)	0.72	0.006 ± 0.069 (−0.030 to 0.043)	−0.128 to 0.140	19.4	0.498 (0.006–0.793)	0.665 (0.011–0.884)
AH	0.664 ± 0.091 (0.615–0.712)	0.681 ± 0.061 (0.649–0.714)	0.673 ± 0.062 (0.639–0.706)	0.455	−0.018 ± 0.091 (−0.066 to 0.031)	−0.197 to 0.162	11.3	0.307 (−0.212–0.688)	0.469 (−0.539–0.815)
MD (10^−3^ mm^2^/s)									
PT	1.158 ± 0.066 (1.123–1.193)	1.182 ± 0.056 (1.152–1.212)	1.170 ± 0.053 (1.142–1.198)	0.141	−0.024 ± 0.061 (−0.056 to 0.009)	−0.143 to 0.096	5.2	0.483 (0.036–0.778)	0.651 (0.069–0.875)
DC	1.171 ± 0.059 (1.140–1.202)	1.169 ± 0.060 (1.138–1.201)	1.170 ± 0.047 (1.145–1.195)	0.933	0.002 ± 0.073 (−0.038 to 0.041)	−0.142 to 0.146	5.1	0.245 (−0.305–0.657)	0.393 (−0.877–0.793)
AH	1.069 ± 0.085 (1.023–1.114)	1.086 ± 0.054 (1.057–1.115)	1.078 ± 0.056 (1.048–1.107)	0.438	−0.018 ± 0.088 (−0.064 to 0.029)	−0.190 to 0.155	6.5	0.247 (−0.274–0.653)	0.396 (−0.753–0.79)
AD (10^−3^ mm^2^/s)									
PT	2.666 ± 0.136 (2.593–2.738)	2.710 ± 0.114 (2.649–2.771)	2.688 ± 0.119 (2.624–2.751)	0.042	−0.044 ± 0.080 (−0.087 to −0.002)	−0.200 to 0.112	4.6	0.760 (0.406–0.912)	0.864 (0.578–0.954)
DC	2.812 ± 0.125 (2.745–2.878)	2.818 ± 0.131 (2.749–2.888)	2.815 ± 0.114 (2.754–2.876)	0.822	−0.007 ± 0.115 (−0.068 to 0.055)	−0.232 to 0.218	4.5	0.610 (0.169–0.845)	0.758 (0.289–0.916)
AH	1.880 ± 0.126 (1.812–1.947)	1.897 ± 0.133 (1.827–1.968)	1.888 ± 0.113 (1.828–1.948)	0.592	−0.018 ± 0.128 (−0.086 to 0.051)	−0.268 to 0.233	6.9	0.524 (0.048–0.804)	0.688 (0.092–0.892)

FA = fractional anisotropy; RD = radial or perpendicular diffusivity; MD = mean diffusivity; AD = axial diffusivity; PTs = pyramidal tracts; DCs = dorsal columns; AHs = anterior horns; CI = confidence interval; CV = coefficient of variation; ICC = intraclass correlation coefficient; LOAs = Limits of Agreement; SD = standard deviation.

**Table 4:** Comparison of the pooled results of quantitative measurements of anisotropy and diffusivity in healthy participants (*n* = 16) between Magnetom Skyra and Magnetom Prisma.

Parameter	Values			Bland–Altman Analysis		Within-Participant Coefficient of Variation CV %	Intraclass Correlation Coefficient (ICC)	
	Skyra_pooled_ ± SD (CI 95%)	Prisma_pooled_ ± SD (CI 95%)	*p* Skyra_pooled_ vs. Prisma_pooled_	Difference (D)	LOA		Single (CI 95%)	Average (CI 95%)
FA								
PT	0.580 ± 0.025 (0.567–0.593)	0.586 ± 0.020 (0.576–0.597)	0.104	−0.007 ± 0.015 (−0.015 to 0.002)	−0.036 to 0.023	3.8	0.755 (0.432–0.907)	0.860 (0.603–0.951)
DC	0.605 ± 0.023 (0.593–0.617)	0.611 ± 0.019 (0.600–0.621)	0.219	−0.006 ± 0.017 (−0.015 to 0.004)	−0.039 to 0.028	3.4	0.653 (0.268–0.862)	0.790 (0.422–0.926)
AH	0.414 ± 0.033 (0.396–0.432)	0.404 ± 0.033 (0.386–0.421)	0.239	0.010 ± 0.033 (−0.008 to 0.028)	−0.055 to 0.075	8.1	0.493 (0.037–0.785)	0.661 (0.071–0.88)
RD (10^−3^ mm^2^/s)								
PT	0.412 ± 0.061 (0.380–0.444)	0.411 ± 0.060 (0.380–0.443)	0.924	0.001 ± 0.032 (−0.017 to 0.018)	−0.063 to 0.064	14.6	0.863 (0.649–0.95)	0.926 (0.787–0.974)
DC	0.345 ± 0.060 (0.314–0.377)	0.348 ± 0.058 (0.317–0.379)	0.82	−0.003 ± 0.046 (−0.027 to 0.022)	−0.093 to 0.087	17	0.709 (0.337–0.888)	0.830 (0.505–0.941)
AH	0.646 ± 0.064 (0.612–0.680)	0.673 ± 0.062 (0.639–0.706)	0.092	−0.027 ± 0.060 (−0.059 to 0.005)	−0.144 to 0.090	9.6	0.518 (0.083–0.796)	0.683 (0.152–0.886)
MD (10^−3^ mm^2^/s)								
PT	1.130 ± 0.052 (1.102–1.158)	1.170 ± 0.053 (1.142–1.198)	0.021	−0.040 ± 0.062 (−0.073 to −0.007)	−0.161 to 0.081	4.6	0.255 (−0.145–0.628)	0.407 (−0.339–0.771)
DC	1.118 ± 0.066 (1.083–1.153)	1.170 ± 0.047 (1.145–1.195)	0.003	−0.052 ± 0.059 (−0.084 to −0.021)	−0.168 to 0.063	4.9	0.341 (−0.093–0.694)	0.509 (−0.206–0.819)
AH	1.051 ± 0.054 (1.022–1.080)	1.078 ± 0.056 (1.048–1.107)	0.095	−0.027 ± 0.060 (−0.059 to 0.005)	−0.144 to 0.091	5.2	0.382 (−0.069–0.72)	0.552 (−0.149–0.837)
AD (10^−3^ mm^2^/s)								
PT	2.566 ± 0.147 (2.488–2.645)	2.688 ± 0.119 (2.624–2.751)	0.011	−0.122 ± 0.168 (−0.211 to −0.032)	−0.450 to 0.207	5.1	0.158 (−0.186–0.54)	0.273 (−0.457–0.702)
DC	2.662 ± 0.191 (2.561–2.764)	2.815 ± 0.114 (2.754–2.876)	0.002	−0.153 ± 0.164 (−0.240 to −0.066)	−0.473 to 0.168	5.6	0.318 (−0.104–0.677)	0.482 (−0.232–0.807)
AH	1.862 ± 0.118 (1.799–1.925)	1.888 ± 0.113 (1.828–1.948)	0.439	−0.026 ± 0.132 (−0.097 to 0.044)	−0.285 to 0.233	6.1	0.349 (−0.163–0.712)	0.518 (−0.389–0.832)

FA = fractional anisotropy; RD = radial or perpendicular diffusivity; MD = mean diffusivity; AD = axial diffusivity; PTs = pyramidal tracts; DCs = dorsal columns; AHs = anterior horns; CI = confidence interval; CV = coefficient of variation; ICC = intraclass correlation coefficient; LOAs = Limits of Agreement; SD = standard deviation.

## Data Availability

In order to safeguard the confidentiality of the participants, the data pertaining to this study are currently withheld from public access. The data can be shared upon reasonable request.

## Supplementary Materials

The following supporting information can be downloaded at: https://www.mdpi.com/article/10.3390/diagnostics15162057/s1, Figure S1: Differences in mean diffusivity (MD) and axial diffusivity (AD) between test and retest in healthy study participants for Magnetom Skyra and Magnetom Prisma. Boxplots showing group differences in MD and AD among healthy study participants (*n* = 16) in pyramidal tracts (PTs) and dorsal columns (DCs) for Magnetom Skyra (A) and Magnetom Prisma (B) and between pooled data of both scanners (C). Data is shown as mean and standard deviation (SD). *p*-values are indicated.; Table S1: MR sequence parameters of spinal DTI. MR examinations were performed using a 32-channel receive-only matrix head coil setup in a supine position. The built-in body coil was applied for spin excitation.

## Abbreviations

The following abbreviations are used in this manuscript:

AD	axial or longitudinal diffusivity
AH	anterior horn
CSF	cerebrospinal fluid
CV	coefficients of variation
DC	dorsal columns
DTI	diffusion tensor imaging
FA	fractional anisotropy
GM	gray matter
ICC	intraclass correlation coefficients
MD	mean diffusivity
PT	pyramidal tracts
RD	radial or perpendicular diffusivity
ROI	region of interest
sDTI	spinal diffusion tensor imaging
T	Tesla
