# Supplementary material for: Test–Retest Reliability and Inter-Scanner Reproducibility of Improved Spinal Diffusion Tensor Imaging
Source: Diagnostics (Basel). 2025 Aug 16;15(16):2057. doi: 10.3390/diagnostics15162057 (PMC12385547; doi:10.3390/diagnostics15162057)
Supplement: Supplementary file 1 [file diagnostics-15-02057-s001.zip › diagnostics-3734356-supplementary.pdf]

Supplementary Table S1. MR sequence parameters of spinal DTI**.** MR examinations were performed using a 32-channel receive-only matrix head coil setup in supine position. The built-in body coil was applied for spin excitation.

Parameter	
Echo Time (TE) [ms]	82
Time of repetition (TR) [ms]	1500
Field of view (FOV) [mm]	100x100
image matrix	128x128
Voxel size [mm]	0.78x0.78x5.0
Slice thickness [mm]	5.0
Band witdh (Hz)	752
Fraction of k-space	6/8
Parallel Imaging (iPAT/GRAPPA)	2
Number of averages	32

FOV = Field of View; GRAPPA = GeneRalized Autocalibrating Partial Parallel Acquisition; Hz = Hertz; PAT = parallel acquisition techniques; TE = Echo Time; TR = Time of repetition.
