# Supplementary figures and images for: Test–Retest Reliability and Inter-Scanner Reproducibility of Improved Spinal Diffusion Tensor Imaging
Source: Diagnostics (Basel). 2025 Aug 16;15(16):2057. doi: 10.3390/diagnostics15162057 (PMC12385547; doi:10.3390/diagnostics15162057)

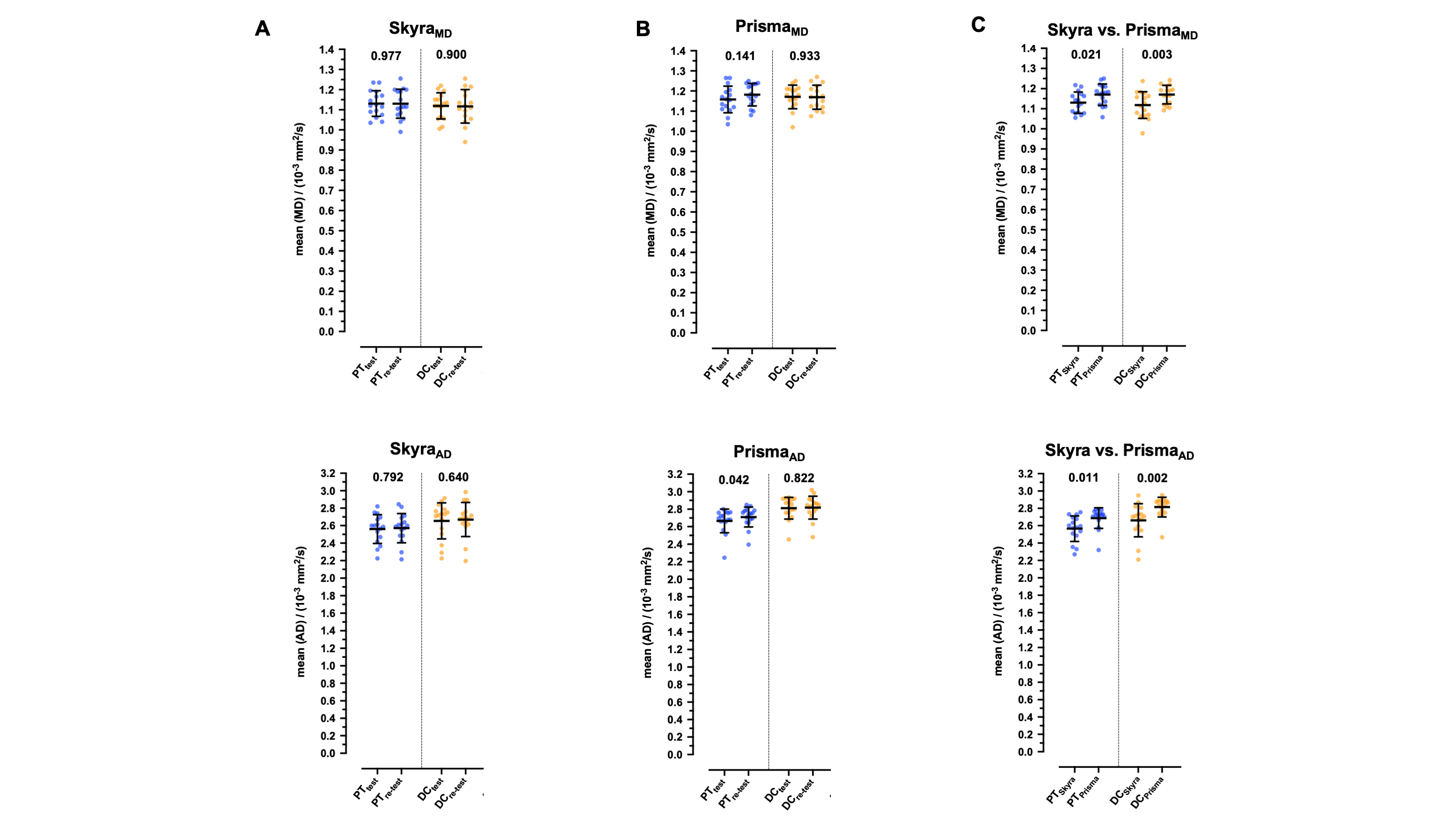

Supplement: Supplementary file 1 [file diagnostics-15-02057-s001.zip › diagnostics-3734356-supplementary.tiff]
